# Preventive effect of *Lactobacillus johnsonii* YH1136 against uric acid accumulation and renal damages

**DOI:** 10.3389/fmicb.2024.1364857

**Published:** 2024-04-16

**Authors:** Xingting Zhang, Junliang Jiang, Jinge Xin, Ning Sun, Zhifang Zhao, Baoxing Gan, Yi Jiang, Xuemei Gong, Hao Li, Hailin Ma, Xueqin Ni, Yu Chen, Yang Bai, Hesong Wang

**Affiliations:** ^1^Guangdong Provincial Key Laboratory of Gastroenterology, Department of Gastroenterology, Institute of Gastroenterology of Guangdong Province, Nanfang Hospital, Southern Medical University, Guangzhou, China; ^2^Baiyun Branch, Nanfang Hospital, Southern Medical University, Guangzhou, China; ^3^Animal Microecology Institute, College of Veterinary Medicine, Sichuan Agricultural University, Chengdu, Sichuan, China; ^4^Department of Gastroenterology, National Institution of Drug Clinical Trial, Guizhou Provincial People’s Hospital, Medical College of Guizhou University, Guiyang, Guizhou, China; ^5^Plateau Brain Science Research Center, Tibet University, Lhasa, Tibet, China; ^6^Guangzhou Beneco Biotechnology Co. Ltd., Guangzhou, China

**Keywords:** hyperuricemia, probiotic, 16S rRNA sequencing, gut microbiota, gut-kidney axis

## Abstract

**Background:**

Hyperuricemia (HUA) is a prevalent metabolic disorder whose development is associated with intestinal microbiota. Therefore, probiotics have emerged as a potential and safe approach for lowering uric acid (UA) levels. However, the underlying mechanisms of many effective probiotic strains remain unknown.

**Methods and results:**

C57BL/6 mice were randomly divided into two groups: control and model groups. The model group received 12 weeks of potassium oxonate. Through 16s sequencing we found that HUA resulted in a significant decrease in the total diversity of all intestinal segments. When each intestinal segment was analyzed individually, the reduction in diversity was only significant in the cecum and colon sections. RDA analysis showed that lactobacilli in the rat colon exhibited a strong correlation with model group, suggesting that *Lactobacillus* may play an important role in HUA. Consequently, the preventive effects of *Lactobacillus johnsonii* YH1136 against HUA were investigated. C57BL/6 mice were randomly divided into three groups: control, model and YH1136 groups. The results showed that administering *Lactobacillus johnsonii* YH1136 effectively reduced serum UA levels *in vivo* by inhibiting hepatic xanthine oxidase (XOD) activity and promoting renal ABCG2 transporter expression. Moreover, supplementation with *Lactobacillus johnsonii* YH1136 significantly ameliorated pathological damage in the kidney and liver, thereby reducing UA accumulation.

**Conclusion:**

Hyperuricemia is accompanied by an altered composition of multiple gut bacteria, of which *Lactobacillus* is a key genus. *Lactobacillus johnsonii* YH1136 may ameliorate renal involvement in HUA via the gut-kidney axis.

## Introduction

The global incidence of hyperuricemia (HUA) continues to rise due to lifestyle changes associated with rapid economic development (Choi, 2010; Major et al., 2018). Over the last century, both men and women have experienced an increase in average serum uric acid (UA) levels (Chen-Xu et al., 2019). For example, in 2019, the prevalence of HUA among Chinese adults reached a staggering 14%, indicating a trend of younger age of onset and higher rates of UA accumulation than those in previous years (Zhang et al., 2021). UA in the human body can originate from endogenous and exogenous sources. Endogenous UA is mainly produced through cellular apoptosis and metabolism (Hyndman et al., 2016), whereas exogenous UA arises from environmental factors, such as consuming seafood, beer, and foods high in oil and sugar (Choi et al., 2004; Aihemaitijiang et al., 2020; Keenan, 2020). UA is mainly excreted from the body through the kidneys and intestines via specific transporter proteins (Ikarashi et al., 2013). However, due to the absence of uricase in humans, UA cannot be directly converted into urea for excretion, resulting in increased UA production and reduced excretion. This imbalance leads to the abnormal accumulation of UA in the body, resulting in hyperuricemia. Although there is no consensus on the definition of hyperuricemia, it is typically diagnosed when serum UA levels exceed 7.0 mg/dL in men and 6.0 mg/dL in women (Ejaz et al., 2020). Moreover, while most patients with HUA remain asymptomatic, the formation of urate crystals can trigger a systemic inflammatory response and the formation of crystalline deposits, which could progress into gout and cause irreversible joint damage. HUA has also been identified as a risk factor for various diseases, such as atherosclerosis, type 2 diabetes, hyperlipidemia, chronic kidney disease, and Alzheimer’s disease (Mortada, 2017; Jayachandran and Qu, 2021; Latourte et al., 2021; Yanai et al., 2021). Given this association, investigating the pathogenesis of HUA and its underlying mechanisms is critical for developing effective prevention and treatment strategies.

The significant role of intestinal microbiota in human health has gained widespread acceptance, leading to an increasing number of studies exploring its connection to various diseases (Cani, 2018). In addition, bidirectional influences between diseases and an imbalance in the intestinal microbiota have been widely observed. In various diseases, disruptions to the intestinal microbiota can cause substantial damage to the intestinal barrier, resulting in the transport of harmful substances from the intestine to other parts of the body (Schmidt et al., 2018; Zhou et al., 2020; Frost et al., 2021). In contrast, a balanced intestinal microbiota lowers disease risk by promoting the colonization of beneficial bacteria, secreting various metabolites, and regulating effector molecule expression (Hand et al., 2016; Agus et al., 2018; Makki et al., 2018). For example, Pan et al. found that disruption in intestinal flora led to abnormal metabolic function of intestinal microorganisms, and potentially induced hyperuricemic nephropathy (Pan et al., 2020). Similarly, Song et al. found that altered intestinal flora composition could contribute to HUA in Uox-KO mice (Song et al., 2022). These findings highlight a strong relationship between intestinal flora and hyperuricemia.

Probiotics have become a popular potential treatment for UA accumulation. Adequate probiotic supplementation can play a regulatory role through the complex interactions of intestinal flora, thereby reducing intestinal flora disorders, ensuring the relative balance of the intestinal microenvironment, and ultimately contributing to the maintenance of a healthy state of the organism (Butel, 2014; Hill et al., 2014). Certain probiotics possess inherent anti-inflammatory properties, while others can play an anti-disease role by regulating intestinal microbiota imbalance and improving intestinal barrier integrity, thereby enhancing immunity (Zheng et al., 2020; Di Luccia and Colonna, 2022; Ragan et al., 2022).

The timing and method of intervention for HUA remain controversial, and their application is limited due to side effects from drug treatment (Chales, 2019; Mehmood et al., 2019; Petreski et al., 2020; Chien et al., 2022). As safer alternatives, probiotics hold great potential for preventing and treating hyperuricemia. Various probiotics have been shown to facilitate UA excretion and reduce hyperuricemia (Xiang et al., 2019). For example, *Limosilactobacillus fermentum* JL-3 can degrade purines, thereby regulating disordered intestinal flora and inhibiting uric acid accumulation (Wu et al., 2021). Similarly, *Lactobacillus paracasei* regulates the expressions of enzymes, such as adenosine deaminase, XOD, and related transporter expression, resulting in reduced UA accumulation *in vivo* (Cao et al., 2022). However, further exploration of effective probiotic strains and understanding their underlying mechanisms of action against HUA are needed, including clarifying many scientific questions regarding how the intestinal microbiota influences HUA. For example, the specific intestinal segment which is most affected during HUA development has yet to be determined, and the identification of symbiotic bacteria with probiotic potential is needed.

Therefore, in this study, the entire intestinal segments of HUA rats were analyzed in detail and based on the results, Lactobacillus johnsonii YH1136 was selected for further study to provide a scientific basis for its use as a potential treatment for HUA. he objectives of the study are as follows: (1) To assess microbiota alterations in each intestinal segment in an HUA rat model to determine the most affected segment and identify potential probiotic strains, and (2) to provide evidence supporting the potential of identified key flora as possible treatments for HUA.

## Materials and methods

### *Lactobacillus johnsonii* YH1136 culture

We isolated *Lactobacillus johnsonii* YH1136 (CCTCC M 20221116) from the feces of a healthy Tibetan girl in Nagchu, Tibet Autonomous Region, then cultured in de Man, Rogosa, and Sharpe (MRS) broth at 37°C. The bacterial cell count was determined using the plate count method, which involved performing a 10-fold gradient dilution of the bacterial solution with PBS. A dilution gradient of 7–5 was selected, and 10 μL drops of the diluted solution were aspirated onto MRS agar medium. The process was repeated three times. The MRS agar medium was then incubated at 37°C for 24 h before counting the bacteria colonies. Finally, *Lactobacillus johnsonii* YH1136 broth was centrifuged at 3000 rpm, 4°C, for 15 min, and then washed thrice with saline. The bacteria were resuspended in saline (pH 7.0) for experimental use. The suspension concentration was 1 × 10^9^ CFU YH1136/mL (daily gavage dose: 0.2 mL/rat) (Xin et al., 2014).

### Development of the HUA rat model

Specific-pathogen-free SD rats (8-week-old, 180–220 g) were purchased from Si Pei Fu Biotechnology Co., Ltd. (Beijing, China). The rats were housed in a controlled environment at a temperature of 22 ± 2°C under a 12 h light-dark cycle, and fed a standard diet with water *ad libitum*. Rats were given a 1-week acclimatization period before the experiment began. In the first part of the experiment, 16 rats were randomly assigned into two groups of 8 rats each: control and hyperuricemia. The HUA and control groups were given potassium oxonate (dose: 100 mg potassium oxonate/kg body weight, dissolved in 0.2 mL 0.5% CMC) or an equivalent dose of Carboxymethylcellulose sodium (CMC) for 12 weeks, respectively. In the second part, 24 rats were randomly divided into the control, hyperuricemia, and YH1136 groups. The HUA and YH1136 groups were treated similarly to the HUA group of the first experiment, while the control group was handled comparably to the control group in the first part of the study. After administering potassium oxonate by gavage for 1 h, the YH1136 group was gavaged with 0.2 mL of 10^7^ CFU YH1136 bacterial solution, while the other two groups were gavaged with equivalent saline amounts. The experiment lasted 12 weeks. All the animal experiments were performed in accordance with the guidelines for the care and use of laboratory animals approved by the Institutional Ethics Committee (approval number: SYXKchuan2019-187).

### Sample collection

On the 84th day of the experiment, all rats were immediately executed by cervical dislocation according to the guidelines of the animal care facility. Serum samples were collected and stored at 4°C overnight to allow precipitation. The samples were then centrifuged at 3000 × rpm for 25 min at 4°C. The resulting supernatant was carefully aspirated and stored at −80°C. Subsequently, kidney, liver, and intestinal segments were collected. The intestinal contents were initially stored on ice and immediately transferred to −80°C. Tissues were washed with ice-cold RNase-free water and blotted with absorbent paper, after which they were immediately stored at −80°C. Some kidney and ileal tissues were preserved in paraformaldehyde for future use in pathological sectioning.

### Biochemical testing

Serum UA (BC1365) levels and blood urea nitrogen (BUN) (BC1535) levels were determined according to the instructions. The malondialdehyde levels in rat kidney and liver tissues were determined using a commercial kit provided by Solarbio Technology (Beijing, China) following the provided assay method. The expression levels or activities of interleukin-1β (IL-1β) (H002-1-2), interleukin-6 (IL-6) (H007-1-1), interleukin-10 (IL-10) (H009-1-2), interleukin-18 (IL-18) (H015-1-2) and Tumor Necrosis Factor-α (TNF-α) (H052-1-2) in rat serum and kidneys were determined using ELISA kits (Nanjing Jiancheng Bioengineering Institute, China) according to the manufacturer’s instruction.

### Histological testing

The kidney and hepatic tissues were dehydrated in wax and embedded in paraffin. Serial sections of the tissue samples were cut at a thickness of 5 μm, then stained with hematoxylin and eosin (HE) and Masson and observed under light microscopy (Olympus, Tokyo, Japan).

Immunohistochemical experiments were performed as previously described (Sun et al., 2020). Kidney biopsy sections were fixed in Michel’s fixative or 10% neutral buffered formalin and subjected to standard staining procedures using rabbit anti-mouse ABCG2 antibody (1:500, 27286-1-AP, Proteintech, China). The stained sections were examined under a light microscope.

### Real-time quantitative PCR (RT-qPCR) detection of mRNA expression levels

Total RNA from kidney and liver samples was isolated using the E.Z.N.A.^®^ Total RNA Kit (OMEGA BioTek, Doraville, GA, USA), according to the manufacturer’s instructions. The quantity and quality of the isolated RNA were assessed using agarose gel electrophoresis, and the absorbance ratio was measured at 260 and 280 nm. The isolated RNA was synthesized as first-strand complementary DNA (cDNA) using the Prime Script TM RT kit and a gDNA eraser (Thermo Scientific, Waltham, MA, USA), following the manufacturer’s instructions. The resulting cDNA products were stored at −20°C for subsequent gene expression analysis. RT-qPCR was performed using the LightCycle^®^ 96 Real-Time System (Boehringer Mannheim GmbH, Germany) and SYBR Green Supermix (Bio-Rad, Hercules, CA, USA). The RT-qPCR reaction mixture contained cDNA product (1 μL), forward primer (1 μL), upstream primer (1 μL), SYBR Green Supermix (5 μL), and sterile deionized water (2 μL). The RT-qPCR procedure consisted of an initial 50°C for 2 min, followed by 95°C for 5 min, then denaturation at 95°C for 10 s, and annealing at the optimal temperature for 30 s, for 40 cycles. The final melting points were profiled to determine the purity of the PCR products. The target gene expression levels at the mRNA level were determined using the above procedure. The relevant target gene expression levels were calculated as fold changes after β-actin normalization using the 2-ΔΔCq relative abundance method in Microsoft Excel. In the kidney groups, mRNA levels of ABCG2, GLUT9, OAT1, OAT3, XOD, IL-6, IL-1β, TNF-α, IL-10, IL-18, and β-actin were measured. In the liver groups, mRNA levels of XOD and actin were detected. Table 1 shows the forward and reverse primer sequences used in this study, which were synthesized by Hangzhou Youkang Biotechnology Company Limited (Hangzhou, Zhejiang, China).

**TABLE 1:** Primers for qPCR.

Gene	Sequence (5′-3′)
β-actin	Forward CGCGTCCACCCGCGAG
	Reverse CCTGGTGCCTAGGGCG
XOD	Forward TGACTGCGGATGAGTTGGTC
	Reverse GTCCCACATAGCCCCAACTT
ABCG2	Forward CCTCGATATGGCTTCACAGCTT
	Reverse GGCCACATGATTCCTCCACA
GLUT9	Forward GCCGAGGAGGACAAAGAACTG
	Reverse CACCATCAGTGTTCCCACCA
OAT1	Forward GCCATACGATCATTCGCACC
	Reverse GGCCTGTCTGCCGAATCA
OAT3	Forward CCTCGGAATAGCCAACCACA
	Reverse GCACACCAAGTCCCACTCTAT
IL-1β	Forward ACTCGTGGGATGATGACGAC
	Reverse GTTTGGGATCCACACTCTCCAG
IL-6	Forward GACTTCCAGCCAGTTGCCTT
	Reverse ATTGCCATTGCACAACTCTTTT
IL-10	Forward GGGAGAGAAGCTGAAGACCC
	Reverse ACACCTTTGTCTTGGAGCTTATTA
IL-18	Forward TATCGACCGAACAGCCAACG
	Reverse GATAGGGTCACAGCCAGTCC
TNF-α	Forward ATCCGAGATGTGGAACTGGC
	Reverse TCCGCTTGGTGGTTTGCTAC

### DNA isolation and 16S rRNA amplicon sequencing

Total genomic DNA samples were extracted using the E.Z.N.A.^®^ Stool DNA Kit (Omega BioBio-Tek, Norcross, GA, USA) following the manufacturer’s instructions. The samples were stored at −20°C for further analysis. The quantity and quality of extracted DNA were measured using a NanoDrop NC2000 spectrophotometer (Thermo Fisher Scientific, Waltham, MA, USA) and agarose gel electrophoresis, respectively. The bacterial 16S rRNA gene V3–V4 region was amplified using PCR with the forward primer 338F (5′-ACTCCTACGGGAGGCAGCA-3′) and reverse primer 806R (5′-GGACTACHVGGGTWTCTAAT-3′). To enable multiplex sequencing, sample-specific 7-bp barcodes were incorporated into the primers. The PCR reaction contained 5 μL of buffer (5 ×), 0.25 μL of Fast Pfu DNA Polymerase (5 U/μL), 2 μL (2.5 mM) of odNTPs, 1 μL (10 μM) each of the forward and reverse primers, 1 μL of DNA template, and 14.75 μL of ddH_2_O. The thermal cycling process included initial denaturation at 98°C for 5 min, followed by 25 cycles consisting of denaturation at 98°C for 30 s, annealing at 53°C for 30 s, and extension at 72°C for 45 s, with a final extension of 5 min at 72°C. PCR amplicons were obtained using Vazyme VAHTSTM DNA Clean Beads (Vazyme, Nanjing, China) and quantified using the Quant-iT PicoGreen dsDNA Assay Kit (Invitrogen, Carlsbad, CA, USA) according to manufacturer instructions. After the individual quantification step, the amplicons were pooled in equal proportion, and paired-end 2 × 250 bp sequencing was performed using the Illumina MiSeq platform at Shanghai Personal Biotechnology Co., Ltd. (Shanghai, China).

### Sequence analysis

Microbiome bioinformatics analysis was performed using QIIME2, with minor modifications, based on the official tutorials^1^ (Bolyen et al., 2019). Briefly, raw sequence data were demultiplexed using the Demux plugin and primer cutting using the cutadapt plugin (Martin, 2011). The sequences were then quality-filtered, denoised, and merged. The chimeras were then removed using the DADA2 plugin (Callahan et al., 2016). The non-singleton amplified sequence variants (ASVs) were aligned with MAFFT and used to construct a phylogenic tree using fasttree2 (Katoh et al., 2002; Price et al., 2009). All samples were rarefied to 309,024 sequences for rarefaction of the microbiota, and ASV richness was observed (Shannon, 1948). Taxonomy was assigned to ASVs using the classify-sklearn naïve Bayes taxonomy classifier in the feature-classifier plugin against the SILVA Release 138 database (Quast et al., 2013; Bokulich et al., 2018). We sequenced the microbiota of the duodenum, jejunum, ileum, cecum, and colon in the two groups of rats.

### Bioinformatics and statistical analysis

In-depth sequence analyses were primarily performed using the QIIME2 and R packages (v3.2.0). Alpha diversity indices, such as the richness estimator and Shannon diversity index, were calculated based on an even abundance table and presented as boxplots. To standardize the number of filtered ASV sequences, the trimmed average M method edgeR package was utilized (Robinson et al., 2010). Beta diversity analysis, or the structural variation in microbial communities across samples, was explored using the Jaccard and Bray–Curtis metrics. The result was visualized via principal coordinate analysis (PCoA) (Bray and Curtis, 1957; Lozupone et al., 2007). The significant differences in microbiota structure among the groups were assessed using the Wilcoxon test using QIIME2 (McArdle and Anderson, 2001; Anderson et al., 2006). The taxonomic composition and abundance were visualized as stacked histograms using the R software. The focus was on showing the distribution at the phylum and genus levels with relative abundances of greater ≥1% in different groups, while those with relative abundances <1% were grouped as “other.” A pairwise Wilcoxon test was used to assess significant differences in microbial taxa (including phyla and genera) between each group.

Additionally, Linear Discriminant Analysis Effect Size (LEfSe) was used to identify differentially abundant taxa across groups (Segata et al., 2011). Linear discriminant analysis was conducted with the standard test, including Kruskal–Wallis and Wilcoxon tests, to determine the statistical significance of the results. For each group, *p*-values (factorial Kruskal-Wallis test) and discriminant trait thresholds (log LDA scores) calculated for microbial taxa with *p* < 0.05 and LDA scores >2, respectively, will be grouped into resultant plots for presentation. Significant indicator species (*p* < 0.05) results were displayed in the bipartite network, and the correlation between ASV and one or more different ASVs was labeled. The bipartite network was constructed from a Fruchterman–Reingold layout with 10^4^ permutations. Key microorganisms from different groups in each intestinal segment were identified using indicator species analysis and then shown in a dichotomous network. These indicator species were obtained based on their statistical significance (*p* < 0.05) and marked correlations between ASV and one or more ASVs. The network was constructed from a Fruchterman–Reingold layout with 104 alignments.

The indicator species identified in different gut segments were clustered into separate indicator genera. Redundancy analysis (RDA) was used to rank the sample and indicator genera in descending order to form fitted values for multiple linear regressions between the response and explanatory variable matrices. The variation in species multiplicity was decomposed into variances associated with the indicator genera, allowing exploration of the relationship between the community composition of each gut segment while considering the presence of the indicator genera. Optimal pairings in the screening model were calculated using variance inflation factors and antecedent selection. Finally, the ranking results were viewed on a type-II scale, and RDA plots were drawn.

The pseudo-count value of SparCC was set to 0.01. The correlation coefficient cut-off value of 0.5 was determined using a random matrix theory-based method implemented in R. Based on the correlation coefficient, a co-oc occurrence network was constructed, where nodes represented ASVs, and edges represented correlations between ASVs. The network was visualized using the R package. The key ASVs were reflected in the SparCC co-occurrence network based on the analysis of the indicator species, forming a network of indicator species and their companion species. A greedy optimized modular algorithm was used within the co-occurrence network to identify and visualize the community modules associated with the indicator species. Further, all species in the community modules were extracted for statistical analysis (Clauset et al., 2004).

### Statistical analysis

All results are presented as mean ± standard deviation. Normality was assessed using the Shapiro–Wilk normality test. If the data conformed to a normal distribution, they were log-transformed for analysis. Non-normal distributed data were analyzed using the Kruskal–Wallis and Wilcoxon tests. All data were evaluated using a one-way analysis of variance (ANOVA), followed by a *post hoc* least significant difference (LSD) test. Statistical significance was defined as *p* < 0.05. Statistical data analysis was performed using IBM SPSS Statistics 25.

## Results

### A stable model of chronic hyperuricemia was established

**FIGURE 1:**
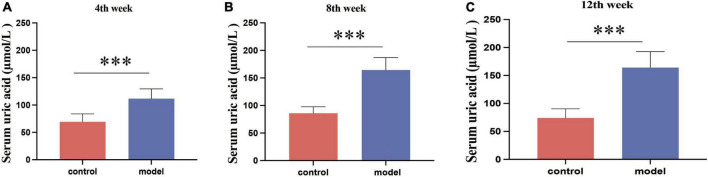
Serum uric acid results in rats at week 4 **(A)**, week 8 **(B)**, and week 12 **(C)**. Administration of potassium oxyzincate caused an increase in uric acid at week 4 and persisted until week 12. Data are expressed as mean ± standard deviation (*n* = 8). Differences between groups are indicated by asterisks (ns > 0.05, **p < 0.01).

During the 12-week experiment period, blood was collected to assess serum UA levels in mice. Figure 1 shows that HUA mice group exhibited significantly higher serum UA levels than those in the control group at week 4 (*p* < 0.001). HUA rats maintained elevated UA levels (*p* < 0.001) throughout the experiment, which stabilized at approximately 200 μmol/L. These findings support the successful establishment of an HUA rat model.

### 16S rRNA gene sequencing results

A total of 15,118,792 raw reads were obtained from 120 fecal samples, averaging 125,990 raw reads per sample, with an average read length of 450 bp. After undergoing quality filtering, denoising, and chimera removal, the accumulated number of ASVs was 1,623,645. After further homogenization and screening, this ultimately resulted in 30,902 ASVs, averaging 2,575 ASVs per sample. The rarefaction curves showed a gradual stabilization of the observed species number, suggesting that the sequencing data were reliable and that the sample had uniform species composition. These findings sufficiently reflect the diversity of the sample, allowing subsequent analyses to be performed.

### HUA causes changes in the gastrointestinal tract (GIT) microbial diversity

**FIGURE 2:**
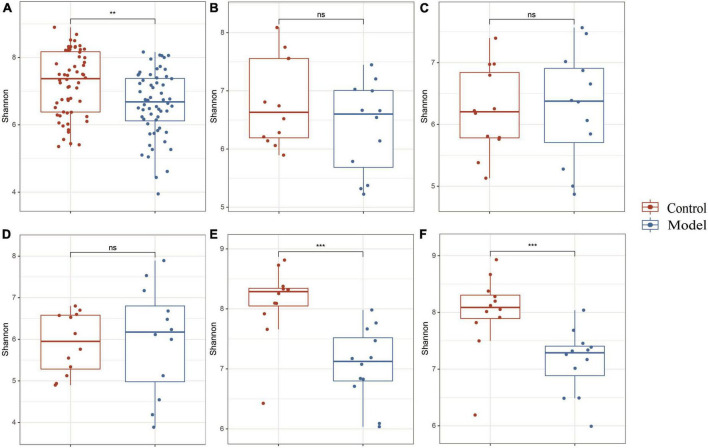
Alpha diversity in different segments of the gastrointestinal tract. The above results are for all the intestinal segments **(A)**, duodenum **(B)**, jejunum **(C)**, ileum **(D)**, cecum **(E)**, and colon **(F)**, respectively. Data are expressed as mean ± standard deviation (*n* = 8). Differences between groups are indicated by asterisks (ns > 0.05, **p < 0.01, ***p < 0.001).

**FIGURE 3:**
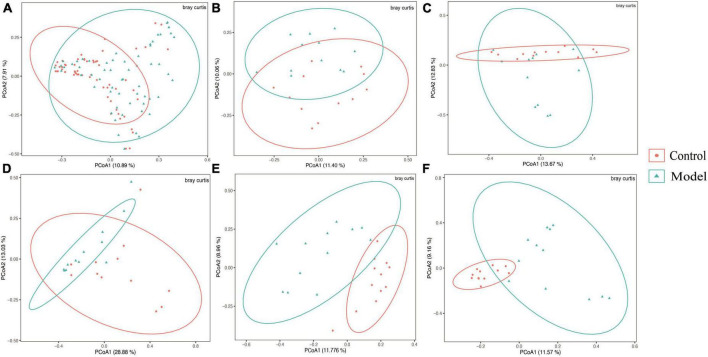
Beta diversity of different gastrointestinal segments based on bray–curtis distance. The above results are for all the intestinal segments **(A)**, duodenum **(B)**, jejunum **(C)**, ileum **(D)**, cecum **(E)**, and colon **(F)**, respectively. Data are expressed as mean ± standard deviation (*n* = 8). Differences between groups are indicated by asterisks (ns > 0.05).

Figure 2A shows that the continuous induction of a high UA model significantly reduced the Shannon diversity index of the rat gastrointestinal tract flora at the entire intestinal segment level (*p* < 0.01). Using Bray–Curtis, beta diversity analysis of the whole intestinal flora showed a significant difference between the two groups, despite a small separation between their clusters (*p* < 0.05) (Figure 3A). Further analysis of the changes in intestinal flora alpha diversity in different intestinal segments revealed that the Shannon diversity indices of the cecal and colonic microbiota were reduced in the HUA group compared to those in the control group (*p* < 0.001) (Figures 2E, F). No significant differences were observed in the Shannon diversity indices of the microbial flora between the two groups in the duodenum, jejunum, and ileum (Figures 2B–D). Moreover, principal coordinate analysis (PCoA) based on Bray-Curtis showed that the cecum and colon samples from the HUA group were clearly separated from those of the control group (Figures 3E, F). However, the samples in the duodenum, jejunum, and ileum were not clearly separated between these two groups (Figures 3B–D).

### Predominant bacteria, classified at the phylum and genus levels, in the GIT of HUA rats

**FIGURE 4:**
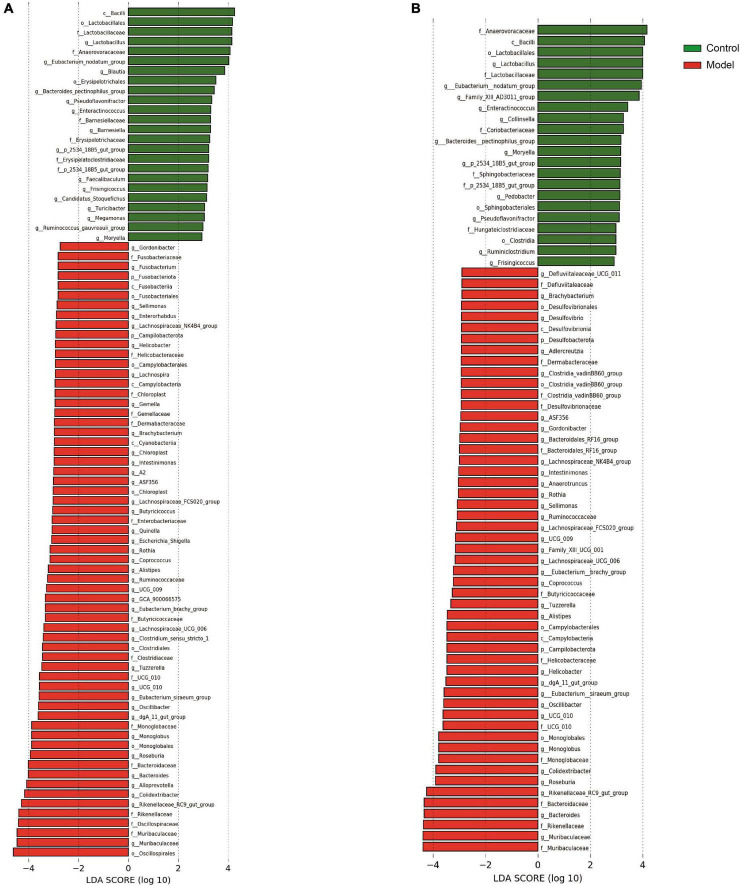
Predominant bacteria with classification from phylum in the cecum **(A)** and colon **(B)**.

Based on the diverse results obtained, the analysis was focused on the composition of the intestinal flora in the colon and cecum. The results of microbiological analysis of the cecum are shown in Figure 4. Approximately 99.77% of the ASVs from the cecum were accurately classified into 13 phyla and 197 genera. Figure 4 shows three phyla and 25 genera with relative abundances >1%. In contrast those of the previous intestinal segments (Supplementary Figure 1), the top three dominant phyla in normal and HUA rats were *Firmicutes*, *Bacteroidota*, and *Actinobacteriota*. The potassium oxonate treatment resulted in limited changes in the cecal microflora composition. However, compared with those in the ileum (Supplementary Figure 4), the following changes occurred in the cecum: *Firmicutes* increased from 54.28 to 73.86%, *Bacteroidota* increased from 1.76 to 22.67%, while *Actinobacteriota* decreased from 30.64 to 2.00% and *Fusobacteriota* decreased from 7.06 to 0%.

The main genera observed at the genus level in the cecum of healthy rats included *Lachnospiraceae*_unidentified (17.32, 6.58 to 26.17%), *Prevotellaceae*_UCG-003 (6.90, 0 to 52.58%), and *Lachnospiraceae*_NK4A136_group (6.83, 1.33 to 19.27%). However, in HUA rats, the abundances of *Prevotella*, *Romboutsia*, and *Lachnospiraceae*_undentified, which formed the three dominant genera, increased from 0.52 to 9.83%, 4.71 to 8.28%, and 0.82 to 55.93%, respectively. Additionally, compared to those of the control, an increase in the abundances of *Prevotella* (from 0.52 to 9.83%), *Lactobacillus* (from 0.61 to 2.12%), and *Romboutsia* (from 4.71 to 8.28%) were observed. Conversely, in the HUA group, the abundances of *Muribaculaceae*, *Rikenellaceae*_RC9_gut_group, and *Bacteroides* decreased from 6.27 to 2.21%, 3.28 to 0.69%, and 2.16 to 0.71%, respectively. Compared to those in the model group, the following changes in the hindgut flora were observed in the control group (Supplementary Figure 5): a significant decrease in the relative abundance of *Romboutsia* (from 50.40 to 8.28%), and increases in the abundances of *Prevotella* (from 0.47 to 9.83%), *Prevotellaceae*_UCG-003 (from 0.19 to 5.70%), and *Lachnospiraceae*_NK4A136_group (from 0.61 to 4.66%). Further, while the advantages at the phylum level were essentially the same, LEfSe analysis at the genus level revealed three genera that had a greater impact on the differences between the two groups: *Lactobacillus*, *Eubacterium nodatum*, and *Blautia*.

Based on the results presented in Figure 4 and Supplementary Figure 6, the composition of the colonic flora was as follows: overall, 98.43% of ASVs were classified into 17 phyla and 237 genera, as the figure shows three phyla and 24 genera. At the phylum level, Firmicutes, Bacteroidota, and Actinobacteriota were dominant in both normal and HUA rats. Notably, the abundance of *Bacteroidota* (from 30.38 to 33.92%) in the HUA group was significantly higher than that in the healthy group. However, the other phyla that exhibited change were mainly low-abundance phyla. Meanwhile, the abundances of *Bacteroidota* and *Actinobacteriota* increased from 22.67 to 33.92% and from 2.00 to 2.66%, respectively, in the ileum of the HUA group (Supplementary Figure 4), while that of *Firmicutes* decreased from 73.86 to 61.84% in the cecum. At the genus level, the dominant genera in the HUA group were *Lachnospiraceae*_unidentified (14.95, 2.85 to 45.86%), *Prevotella* (14.55, 0.25 to 66.29%), and *Alloprevotella* (7.38, 0.12 to 51.26%). Conversely, the dominant genera observed in the healthy group included *Lachnospiraceae*_undentified (13.19, 2.70 to 29.80%), *Prevotellaceae*_UCG-003 (7.24, 0.27 to 50.76%), and *Muribaculaceae* (6.51, 0.92 to 9.75%). Moreover, a significant decrease in the abundances of *Prevotellaceae*_UCG-003 (from 7.24 to 4.91%), *Muribaculaceae* (from 6.51 to 3.40%), *Bacteroides* (from 4.51 to 0.86%), *Rikenellaceae*_RC9_gut_group (from 3.48 to 0.68%), and *Lachnospiraceae*_NK4A136_group (from 4.38 to 2.76%), as well as an increase in the abundances of *Lactobacillus* (from 0.80 to 2.40%) and *Blautia* (from 1.78 to 3.38%) were observed in the HUA group compared to those in the control group. In addition, *Lactobacillus*, *Eubacterium nodatum*, and *Family*XIIIAD3011, which were the top three genera significantly enriched in the HUA group, had the greatest impacts on the differences between the two groups.

### Interaction effect of each intestinal segment with key strains

Based on the diverse results obtained, the analysis was focused on the composition of the intestinal flora in the colon and cecum. The results of microbiological analysis of the cecum are shown in Figure 4. Approximately 99.77% of the ASVs from the cecum were accurately classified into 13 phyla and 197 genera. Figure 4 shows three phyla and 25 genera with relative abundances >1%. In contrast those of the previous intestinal segments (Supplementary Figure 1), the top three dominant phyla in normal and HUA rats were *Firmicutes*, *Bacteroidota*, and *Actinobacteriota*. The potassium oxonate treatment resulted in limited changes in the cecal microflora composition. However, compared with those in the ileum (Supplementary Figure 4), the following changes occurred in the cecum: *Firmicutes* increased from 54.28 to 73.86%, *Bacteroidota* increased from 1.76 to 22.67%, while *Actinobacteriota* decreased from 30.64 to 2.00% and *Fusobacteriota* decreased from 7.06 to 0%.

The main genera observed at the genus level in the cecum of healthy rats included *Lachnospiraceae*_undentified (17.32, 6.58 to 26.17%), *Prevotellaceae*_UCG-003 (6.90, 0 to 52.58%), and *Lachnospiraceae*_NK4A136_group (6.83, 1.33 to 19.27%). However, in HUA rats, the abundances of *Prevotella*, *Romboutsia*, *and Lachnospiraceae*_undentified, which formed the three dominant genera, increased from 0.52 to 9.83%, 4.71 to 8.28%, and 24.18, 0.82 to 55.93%, respectively. Additionally, compared to those of the control, an increase in the abundances of *Prevotella*, *Lactobacillus* (from 0.61 to 2.12%), and *Romboutsia* were observed. Conversely, in the HUA group, the abundances of *Muribaculaceae*, *Rikenellaceae*_RC9_gut_group, and *Bacteroides* decreased from 6.27 to 2.21%, 3.28 to 0.69%, and 2.16 to 0.71%, respectively. Compared to those in the model group, the following changes in the hindgut flora were observed in the control group (Supplementary Figure 5): a significant decrease in the relative abundance of *Romboutsia* (from 50.40 to 8.28%), and increases in the abundances of *Prevotella* (from 0.47 to 9.83%), *Prevotellaceae*_UCG-003 (from 0.19 to 5.70%), and *Lachnospiraceae*_NK4A136_group (from 0.61 to 4.66%). Further, while the advantages at the phylum level were essentially the same, LEfSe analysis at the genus level revealed three genera that had a greater impact on the differences between the two groups: *Lactobacillus*, *Eubacterium nodatum*, and *Blautia*.

Based on the results presented in Figure 4 and Supplementary Figure 6, the composition of the colonic flora was as follows: overall, 98.43% of ASVs were classified into 17 phyla and 237 genera, with the most important 3 phylums and 24 genera displayed as shown in figures. At the phylum level, Firmicutes, Bacteroidota, and Actinobacteriota were dominant in both normal and HUA rats. Notably, the abundance of *Bacteroidota* (from 30.38 to 33.92%) in the HUA group was significantly higher than that in the healthy group. However, the other phyla that exhibited change were mainly low-abundance phyla. Meanwhile, the abundances of *Bacteroidota* and *Actinobacteriota* increased from 22.67 to 33.92% and from 2.00 to 2.66%, respectively, in the ileum of the HUA group (Supplementary Figure 4), while that of *Firmicutes* decreased from 73.86 to 61.84% in the cecum. At the genus level, the dominant genera in the HUA group were *Lachnospiraceae*_undentified (14.95%, 2.85% to 45.86%), *Prevotella* (14.55, 0.25 to 66.29%), and *Alloprevotella* (7.38, 0.12 to 51.26%). Conversely, the dominant genera observed in the healthy group included *Lachnospiraceae*_undentified (13.19, 2.70 to 29.80%), *Prevotellaceae*_UCG-003 (7.24, 0.27% to 50.76%), and *Muribaculaceae* (6.51, 0.92 to 9.75%). Moreover, a significant decrease in the abundances of *Prevotellaceae*_UCG-003 (from 7.24 to 4.91%), *Muribaculaceae* (from 6.51 to 3.40%), *Bacteroides* (from 4.51 to 0.86%), *Rikenellaceae*_RC9_gut_group (from 3.48 to 0.68%), and *Lachnospiraceae*_NK4A136_group (from 4.38 to 2.76%), as well as an increase in the abundances of *Lactobacillus* (from 0.80 to 2.40%) and *Blautia* (from 1.78 to 3.38%) were observed in the HUA group compared to those in the control group. In addition, *Lactobacillus*, *Eubacterium nodatum*, and *Family*XIIIAD3011, which were the top three genera significantly enriched in the HUA group, had the greatest impacts on the differences between the two groups.

### Interaction effect of each intestinal segment with key strains

**FIGURE 5:**
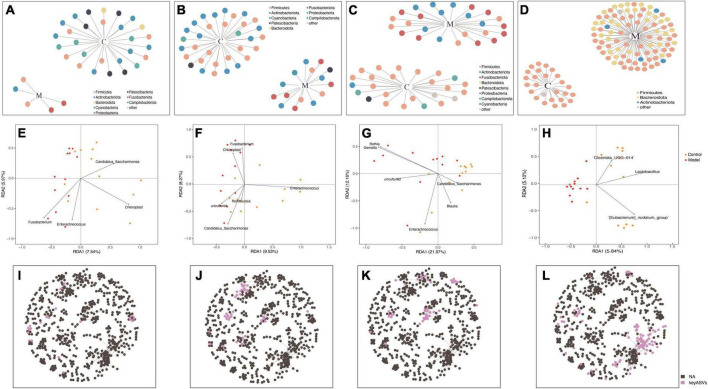
Redundancy analysis (RDA) and co-occurrence network analyses based on indicator species in the duodenum **(A,E,I)**, jejunum **(B,F,J)**, ileum **(C,G,K)**, and colon **(D,H,L)**.

Figures 5A–D depict the microbial patterns observed in the different small intestinal segments (duodenum, jejunum, and ileum), which exhibited similar characteristics. In the control group, the indicator species belonged to the phyla *Firmicutes* and *Actinobacteria*, while in the HUA group, the indicator species were *Firmicutes*, *Actinobacteria*, and *Fusobacteria*. The indicator species in the colonic segment was simpler in structure than that in the small intestine, with the majority belonging to *Firmicutes* in the control group or *Firmicutes*, *Bacteroidota*, and *Actinobacteria* in the HUA group. Notably, no shared indicator species existed in the duodenum, jejunum, ileum, or colon, and the number of indicator species varied significantly across the intestinal segments, indicating unique flora characteristics in each segment.

Redundancy analysis was used to rank the indicator species and samples of each intestinal segment. According to the RDA results (Figures 5E–H), significant differences were observed in the key bacterial genera derived from the screening and clustering of indicator species across different intestinal segments. In the duodenum, the key genera in the control group were *Candidatus_Saccharimonas* and *Chloroplast*, while those in the HUA group were *Enteractinococcus* and *Fusobacterium*. In the ileum, the key genera in the control group were *Candidatus_Saccharimonas*, *Blautia*, and *Enteractinococcus*, while those in the HUA group included *Gemella* and *Rothia*. In the jejunum, Enteractinococcus was the key genera in the control group, while *Candidatus_Saccharimonas* and *Chloroplast* were the key genera of the HUA group. Unlike the small intestinal bowel segments, only the key genera that were positively correlated with controls were present in the colon, namely *Clostridium*_UCG-014, *Lactobacillus* and *[Eubacterium]* nodatumgroup. *Lactobacillus* exhibited a negative correlation with high UA group samples, and the attribute values of each sample were similar. Finally, the key species screened from each intestinal segment in the whole intestine, shown in Figures 5I–L, were present in a co-occurrence network of the whole intestine, and their respective spatial distribution characteristics were identified.

### *Lactobacillus johnsonii* YH1136 reduces uric acid accumulation in HUA rats induced by potassium oxyzincate

**FIGURE 6:**
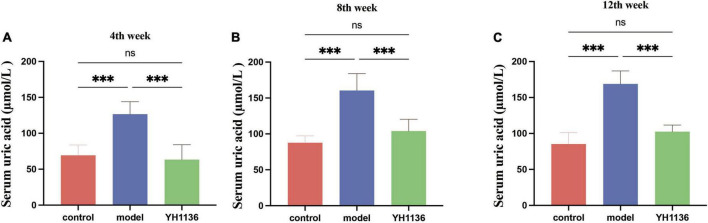
Serum uric acid results in rats at week 4 **(A)**, week 8 **(B)**, and week 12 **(C)**. Gavage of YH1136 slowed the trend of elevated uric acid due to potassium oxyzate. Data are expressed as mean ± standard deviation (*n* = 8). Differences between groups are indicated by asterisks (ns > 0.05, **p < 0.01).

Figure 6 depicts the blood UA levels in the control and experimental rats at different time points. From the fourth week onward, the HUA group exhibited a significantly higher UA levels than those of the control group (*p* < 0.001). Meanwhile, rats in the YH1136 treatment group exhibited significantly reduced serum UA levels compared to those in the control group (*p* < 0.001). Moreover, no significant difference was observed in UA levels between the YH1136 and control groups (*p* > 0.05), and the inhibitory effect of YH1136 on UA accumulation persisted throughout the experimental duration.

### YH1136 can reduce uric acid production and improve liver lesions in hyperuricemia

**FIGURE 7:**
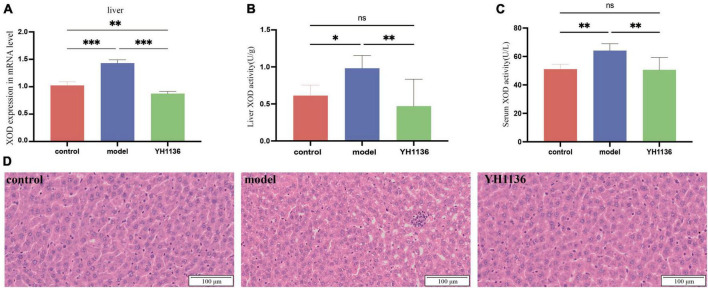
Xanthine oxidase (XOD) mRNA expression level in the liver **(A)**. XOD activity in the serum **(B)** and liver **(C)**. Representative pictures of liver lesions **(D)**. Data are expressed as mean ± standard deviation (*n* = 8). Differences between groups are indicated by asterisks (ns > 0.05, *p < 0.05, **p < 0.01, ***p < 0.001). YH1136 can reduce the expression and activity of XOD in the body, slowing down the accumulation of uric acid and reducing the liver lesions caused by hyperuricemia.

Overall, YH1136 administration reduced XOD production by the liver (Figure 7); the mRNA levels were significantly higher in the HUA group than those in the control (*p* < 0.001) and YH1136 groups (*p* < 0.001). In contrast, the YH1136 group exhibited significantly lower mRNA levels of hepatic XOD than those in the control group (*p* < 0.01) (Figure 7A). Moreover, hepatic XOD activity in the HUA group was significantly higher than that in the control (*p* < 0.05) and YH1136 (*p* < 0.01) groups, while no significant difference was observed between the control and YH1136 groups (*p* > 0.05) (Figure 7B). A similar trend was observed in the serum XOD activity (Figure 7C).

In addition, HE staining of the collected liver tissues showed that HUA induced severe hepatic steatosis with marked infiltration of inflammatory cells within the hepatic lobules, leading to liver injury in rats. In contrast, YH1136 treatment significantly reduced liver damage. These findings reveal the alleviating effect of YH1136 on HUA and suggest its potential therapeutic value for fatty liver disease associated with hyperuricemia (Figure 7D).

### YH1136 promotes kidney uric acid excretion and reduces kidney uric acid accumulation

**FIGURE 8:**
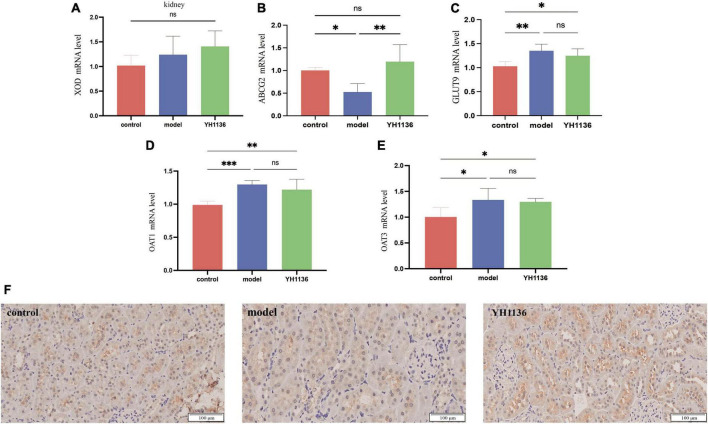
Xanthine oxidase (XOD) expression in mRNA levels at the kidney **(A)**. mRNA expression levels of multiple uric acid transporters in the kidney: ABCG2 **(B)**, GLUT9 **(C)**, OAT1 **(D)**, and OAT3 **(E)**. Immunohistochemical section of the renal uric acid transporter ABCG2. The magnification is 20X **(F)**. Data are expressed as mean ± standard deviation (*n* = 6). Differences between groups are indicated by asterisks (ns > 0.05, *p < 0.05, **p < 0.01, ***p < 0.001).

In the kidneys, no significant differences were observed in XOD mRNA expression among the groups (*p* > 0.05) (Figure 8A), though a series of UA transporters were identified. The mRNA expressions of the GLUT9 (*p* < 0.01), OAT1 (*p* < 0.001), and OAT3 (*p* < 0.05) transporters were significantly higher in the model group than those in the control group; however, no significant differences were observed between the YH1136 and model groups (*p* > 0.05) (Figures 8C–E). Moreover, Figure 8 showed that the mRNA level of the ABCG2 transporter in the model group was significantly lower than that in the control group (*p* < 0.05), whereas that in the YH1136-treated group was significantly higher than that in the model group (*p* < 0.01). However, ABCG2 expression was not significantly different from that in the control group (*p* > 0.05). The immunohistochemical results suggested the same alteration at the protein level, whereas YH1136 reversed the above manifestation, thereby increasing ABCG2 expression (Figure 8F).

### YH1136 alleviates kidney inflammation and reduces hyperuricemic kidney injury

**FIGURE 9:**
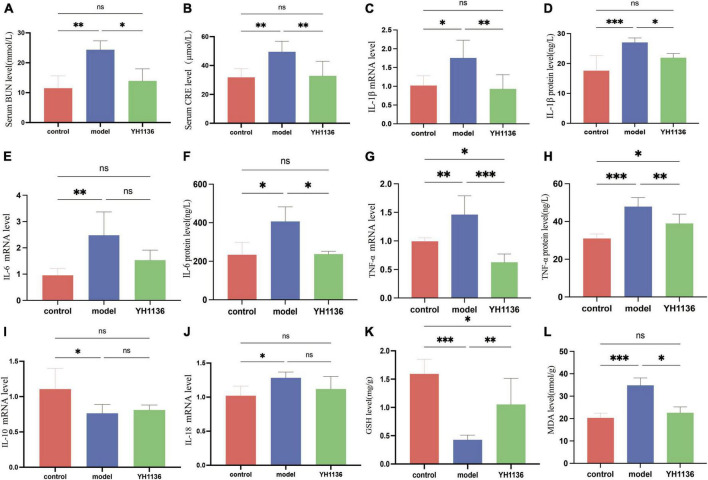
The BUN level in serum **(A)**, CRE level in serum **(B)**, IL-1β mRNA expression level in the kidney **(C)**, IL-1β level in the kidney **(D)**, IL-6 mRNA expression level in the kidney **(E)**, IL-6 level in the kidney **(F)**, TNF-α mRNA expression level in the kidney **(G)**, TNF-α level in the kidney **(H)**, IL-10 mRNA expression level in the kidney **(I)**; IL-18 mRNA expression level in the kidney **(J)**. GSH level in the kidney **(K)**, MDA level in the kidney **(L)**. Data are expressed as mean ± standard deviation (*n* = 8). Differences between groups are indicated by asterisks (ns > 0.05, *p < 0.05, **p < 0.01, ***p < 0.001).

**FIGURE 10:**
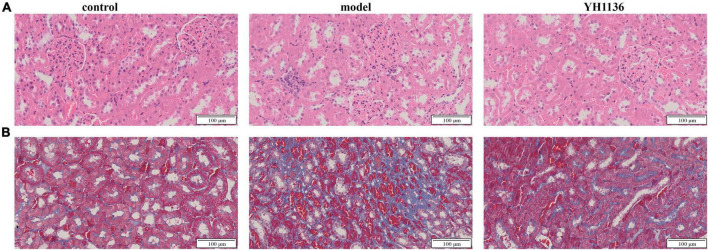
The representative HE pictures in the kidney **(A)**. The representative Masson pictures in the kidney **(B)**. The magnification is 20X.

Since previous results showed a reduction in renal UA accumulation in the YH1136 group, the modulatory effect of YH1136 on hyperuricemic nephropathy was further investigated. First, serum BUN and CRE levels were significantly elevated in the HUA group (*p* < 0.01) (Figures 9A, B), indicating impaired kidney function. Conversely, in the YH1136 group, the levels of the above two indicators were not significantly different from those in the control group (*p* > 0.05) but were significantly lower than those in the HUA group (*p* < 0.05, *p* < 0.01) (Figures 10A, B).

Then, common inflammatory factors were identified. The HUA group exhibited significant increases in IL-1β and IL-6 expressions in the kidney compared to those in the control group (*p* < 0.01 and *p* < 0.05). However, no significant difference was observed between the control and YH1136 groups (*p* > 0.05). These results were consistent with mRNA and protein level results (Figures 9C–F). In the kidneys, the mRNA (*p* < 0.01) and protein levels (*p* < 0.001) of TNF-α were significantly higher in the HUA group than those in the control group. Meanwhile, the YH1136 group exhibited a significant decrease in TNF-α levels (*p* < 0.01), but still differed significantly from the control group (*p* < 0.05). At the mRNA level, YH1136 reduced the expression of TNF-α to a lower level below that in control (*p* < 0.05) (Figures 9G, H). The mRNA level of the anti-inflammatory factor IL-10 was significantly suppressed in the HUA group compared to the control group, whereas the mRNA level of the pro-inflammatory factor IL-18 was significantly elevated in the HUA group (*p* < 0.05). However, YH1136 did not show any significant regulatory effects on these two indices (*p* > 0.05) (Figures 9I, J).

Renal oxidative stress injury was also evaluated. GSH was significantly elevated in the HUA group compared to that in the control group (*p* < 0.001), whereas the YH1136 group effectively suppressed this increase (*p* < 0.05) (Figure 9K). Furthermore, MDA levels were significantly reduced in the YH1136 group compared to those in the model group (*p* < 0.05), but were not significantly different from those in the control group (*p* > 0.05) (Figure 9L). HE staining showed that HUA leads to tubular atrophy, interstitial inflammatory cell infiltration, and interstitial congestion (Figure 10A) while Masson’s staining revealed severe interstitial fibrosis in the HUA group (Figure 10B). However, YH1136 administration shrunk the lesions (Figures 10A, B), suggesting that the kidney plays a crucial role in the therapeutic effect of YH1136 on HUA.

## Discussion

Owing to the overall modern lifestyle, HUA has emerged as the second most common metabolic disease after diabetes, and its onset occurs at younger ages (Liu et al., 2015; Yin et al., 2022). Therefore, the aim of this study was to explore the key strains in the course of HUA, with a view to targeting probiotics that are highly effective in the prevention and treatment of HUA.

Consistent with previous studies, a significant reduction was observed in the overall diversity of the intestinal flora in HUA rats (Wei et al., 2021). Although HUA led to differences in flora diversity in all intestinal segments, the most pronounced differences were found in the cecum and colon, consistent with the results of Han et al. (2023). Therefore, the floral composition in the cecum and colon was comprehensively analyzed, and significant differences in the flora at the genus level were observed between the control and HUA groups. In the cecum, the most distinct genera were *Lactobacillus*, *Eubacterium _ nodatum _ group*, and *Blautia*, whereas in the colon, the differential genera were *Lactobacillus*, *Eubacterium _ nodatum _ group*, and *Family_XIII_AD3011 _ group*. Of these, *Lactobacillus* was a differential indicator in the flora of multiple intestinal segments, consistent with findings in previous studies suggesting that *Lactobacillus* may be a key species in hyperuricemia (Xu et al., 2021). To validate these results, RDA and co-occurrence network analyses were performed to examine the regulatory effects of the indicated flora. Significant associations (*p* < 0.05) were identified between specific ASV changes and distinct intestinal flora patterns, which are crucial in different animal species (Severns and Sykes, 2020). Moreover, no significant co-occurrence patterns were observed among the flora in the cecum. However, a strong association with Lactobacillus was found in the colonic flora, which exhibited the highest fit across all samples. *Lactobacillus* was negatively correlated with samples from the HUA group, which is consistent with findings from previous studies (Xu et al., 2021). Based on the analysis of the entire intestinal flora composition in rats with hyperuricemia, it was found that the colon microbiota exhibited the most significant changes and *Lactobacillus* emerged as a potentially crucial regulator in hyperuricemia. Therefore, a specific strain of *Lactobacillus johnsonii* YH1136, was selected to treat rats with hyperuricemia. This bacterial strain significantly reduced serum UA levels in rats, which encouraged the researcher team to delve deeper into its underlying mechanisms.

The disruption of UA production and excretion *in vivo* contributes to the development of HUA. The liver is the primary site for uric acid production, followed by the kidneys. Furthermore, endogenous and exogenous purines undergo metabolic processes within the liver, resulting in hypoxanthine formation. Subsequently, xanthine oxidase catalyzes the oxidation and reduction of hypoxanthine to form xanthine and, eventually, uric acid (Kimura et al., 2021). In this study, XOD expression in the HUA rats was significantly up-regulated only in the liver, but it was not significantly different in the kidney, which demonstrates the excessive production of UA by the liver during the course of HUA. However, supplementation with YH1136 resulted in a significant reduction in the transcript and protein contents of XOD in the liver, suggesting that YH1136 can inhibit XOD. By inhibiting XOD, YH1136 effectively reduced UA production in the liver and lowered its body level. In addition, YH1136 increased uric acid excretion rates in the body. Based on these results, liver tissue sections from each group were examined and it was found that YH1136 supplementation significantly reduced liver lesions, suggesting its ability to mitigate the severe steatosis observed in the HUA group. The kidneys are the main organs for UA excretion in the body, accounting for approximately 70% of total UA excretion (Wang J. et al., 2022), while the remaining UA is primarily excreted through the intestinal tract (Wang Y. et al., 2022). A crucial factor in the regulation of UA is the activity of transporters. One important transporter is the adenosine triphosphate-binding cassette transporter protein G2 (ABCG2), which belongs to the ABC efflux transporter protein superfamily. ABCG2 is primarily expressed in the apical membrane of the proximal tubule in the kidney (Mao, 2008; Eckenstaler and Benndorf, 2021), where it secretes and excretes UA from the body. In this study, a significant decrease in renal ABCG2 expression was observed during the development of HUA, resulting in impaired UA excretion and subsequent accumulation in the body. However, supplementation with YH1136 effectively reversed this phenomenon and increased renal ABCG2 expression, which facilitated UA excretion from the kidneys. However, no significant differences were observed in ABCG2 expression in the ileum between groups, which suggests that YH1136 supplementation may indirectly promote ABCG2 expression in the kidneys by producing specific metabolites after colonization in the intestine. These metabolites may be transferred to the kidneys to promote ABCG2 expression, thereby increasing UA excretion. In summary, YH1136 administration reduced blood UA levels and inhibited UA accumulation in the body by decreasing hepatic UA production and increasing renal UA excretion.

These findings indicate that the kidneys play a crucial role in HUA pathology. A significant relationship was found between baseline UA levels and the risk of kidney disease, wherein for every 1 mg/dL increase in serum UA levels, the risk of kidney disease in patients increases by 7–11% (Weiner et al., 2008). Further, UA accumulation in the kidneys can result in a condition known as hyperuric acid nephropathy, which is characterized by inflammatory infiltration, UA crystal formation, and kidney fibrosis (Kang et al., 2002; Isaka et al., 2016). Therefore, the relationship between YH1136 expression and kidney injury was investigated. Renal Cre and BUN levels were significantly elevated in the hyperuric acid state, suggesting compromised kidney function. However, supplementation with YH1136 reduced the expression levels of these indicators, suggesting that the damage to renal function was mitigated. Afterward, some common inflammatory factors were examined and it was found that oral administration of YH1136 reduced the elevated renal IL-1β, IL-6, and TNF-α expressions caused by HUA. Additionally, a reduction in inflammatory cell infiltration in the pathological sections of the kidney was observed, suggesting that HUA can lead to oxidative stress in the kidneys, characterized by decreased GSH levels and increased MDA levels. Nevertheless, supplementation with YH1136 significantly improved oxidative stress damage in the kidney. More specifically, a macroscopic examination of the kidney revealed that YH1136 treatment improved glomerular hypertrophy, renal fibrosis, and interstitial congestion lesions associated with hyperuricemic nephropathy. These findings suggest that YH1136 can effectively mitigate renal damage in cases of HUA to some extent, providing a potentially better prognosis for patients with HUA. These findings suggest that *Lactobacillus johnsonii* YH1136 may exert its effect on the kidney through the gut-kidney axis. Moreover, given the slower motility of the colon compared to that of other segments of the intestine, it is speculated that *Lactobacillus johnsonii* YH1136 is more likely to colonize the colon. Based on previous studies (Cheng et al., 2022; Hsu and Tain, 2022), there are two possible mechanisms for the observed effects: (1) supplementation with YH1136 may reduce toxin production via the intestinal flora, thereby mitigating renal involvement, and (2) key metabolites produced by YH1136 in the intestine may enter circulation through the intestine and reach the renal site, where they exert their anti-UA and anti-inflammatory effects, leading to a reduction in renal injury. To elucidate these mechanisms, further studies need to identify the specific substances involved in HUA and explore their precise mechanism of action in the middle of the intestine and kidney.

## Conclusion

In conclusion, the study’s findings reveal that HUA may disrupt the intestinal microbiota composition in different segments of the gastrointestinal tract. Particularly, colonic microbiota play a significant role in HUA development, and *Lactobacillus* is a potentially crucial bacteria for HUA treatment, as supplementation with *Lactobacillus johnsonii* YH1136 effectively reduced UA production, increased UA excretion, inhibited inflammatory response in the kidneys, reduced oxidative stress damage, and significantly improved renal lesions associated with HUA. These findings emphasize the potential significance of *Lactobacillus* in the interaction between HUA and gut microbiota, suggesting the potential ability of *Lactobacillus johnsonii* YH1136 to improve HUA and its enormous potential as an edible strain for treatment. In addition, this study provides a basis for the future development of probiotic formulations that are effective against HUA.

## Data availability statement

The datasets presented in this study can be found in online repositories. The names of the repository/repositories and accession number(s) can be found below: https://www.ncbi.nlm.nih.gov/, PRJNA1059774.

## Ethics statement

The animal study was approved by the Institutional Animal Care and Use Committee of the Sichuan Agricultural University (approval number: SYXKchuan2019-187). The study was conducted in accordance with the local legislation and institutional requirements.

## Author contributions

XZ: Writing – review and editing, Writing – original draft. JJ: Writing – review and editing, Project administration, Formal analysis. JX: Writing – review and editing, Methodology, Conceptualization. NS: Writing – review and editing, Formal analysis, Data curation. ZZ: Writing – review and editing. BG: Writing – review and editing, Formal analysis, Data curation. YJ: Writing – review and editing, Validation, Resources. XG: Writing – review and editing, Project administration. HL: Writing – review and editing, Funding acquisition. HM: Writing – review and editing, Supervision, Funding acquisition. XN: Writing – review and editing, Supervision, Funding acquisition. YC: Writing – review and editing, Software, Resources, Supervision. YB: Writing – review and editing, Supervision, Funding acquisition. HW: Writing – review and editing, Supervision.

## Footnotes

1
https://docs.qiime2.org/
