# Supplementary material for: Preventive effect of Lactobacillus johnsonii YH1136 against uric acid accumulation and renal damages
Source: Front Microbiol. 2024 Apr 16;15:1364857. doi: 10.3389/fmicb.2024.1364857 (PMC11059993; doi:10.3389/fmicb.2024.1364857)
Supplement: Supplementary file 1 [file Table_1.DOCX]

**Preventive effect of Lactobacillus johnsonii YH1136 against uric acid accumulation and renal damages**

**Supplementary Information**

**
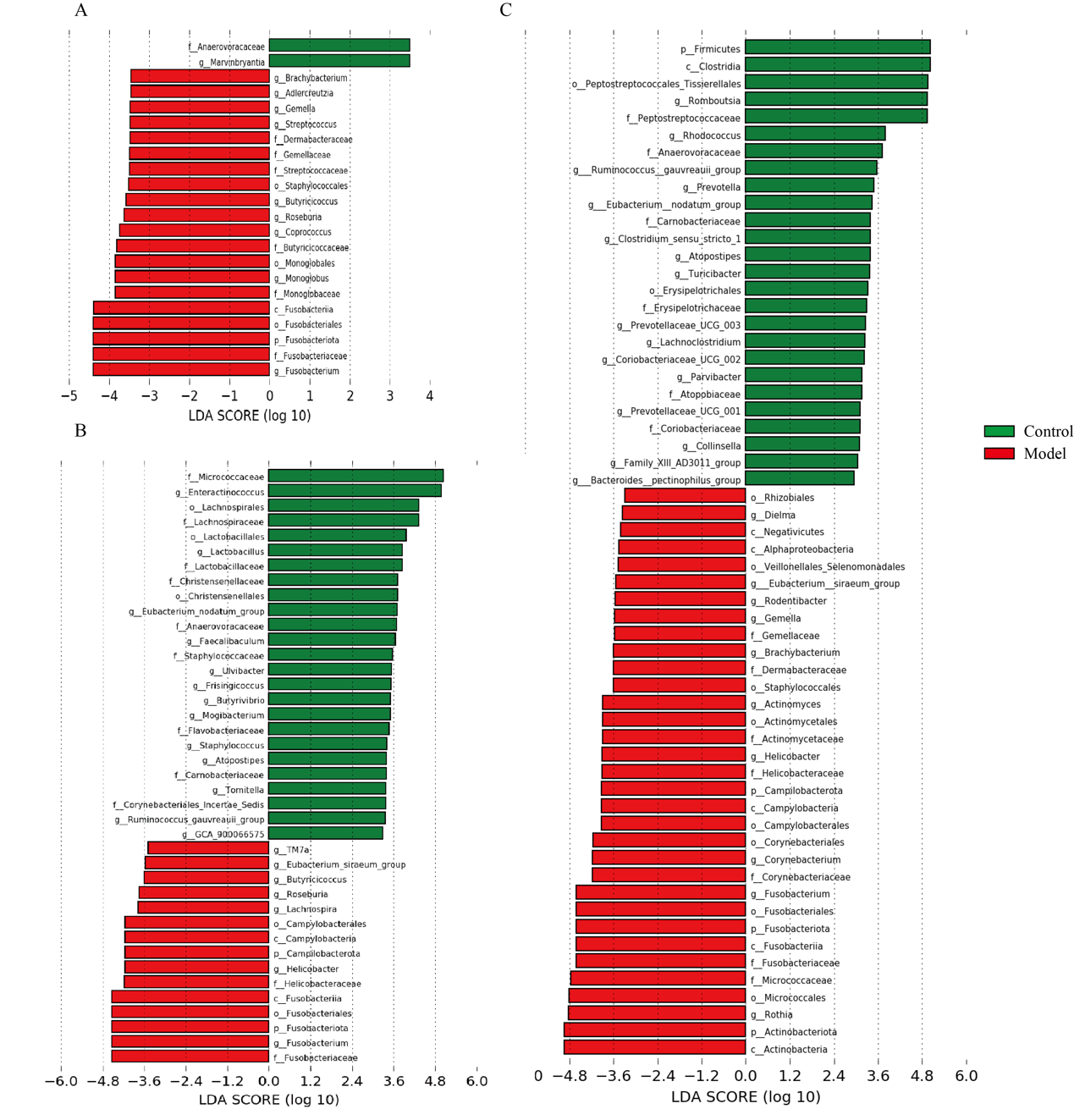
**

**Fig. S1. Linear Discriminant Analysis Effect Size Analysis. Significantly discriminative taxa (Biomarkers) between the control and model groups of mice were displayed by LDA value distribution histogram in Duodenum(A), jejunum(B) and ileum(C). Only taxa meeting the LDA significance thresholds (>4) are shown. Different coloured bars and regions represent different groups.**

**
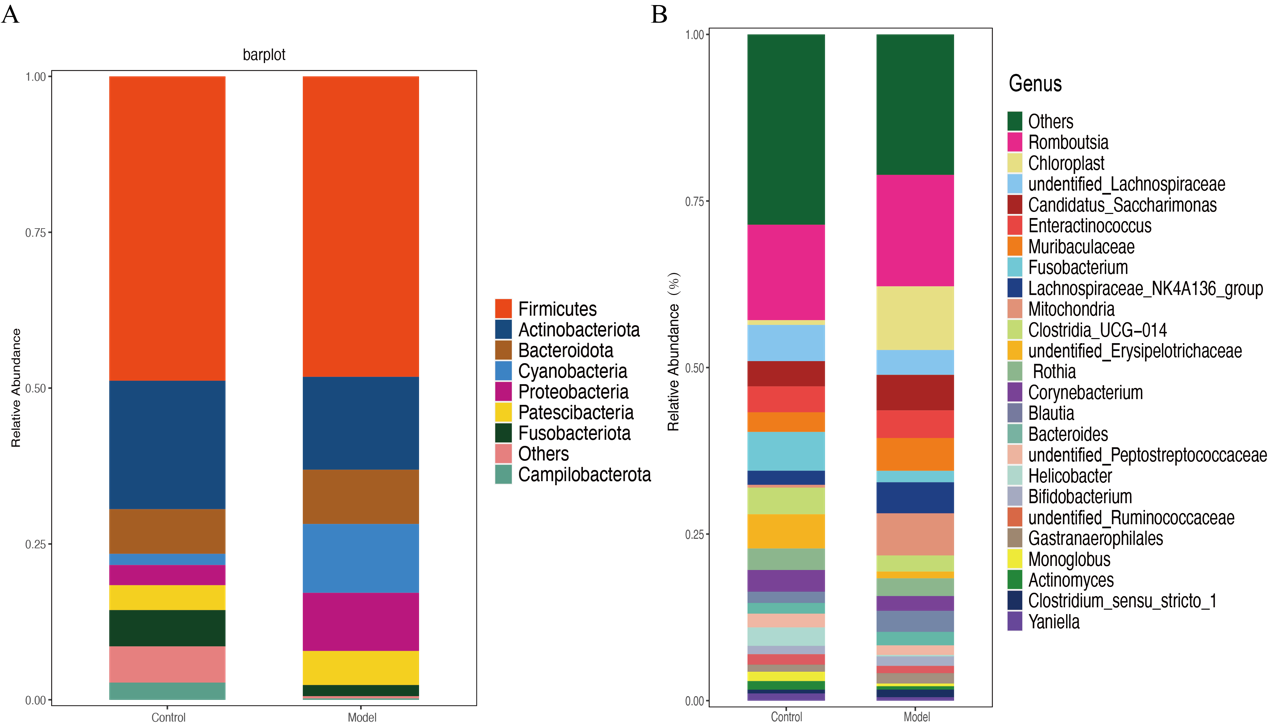
**

**Fig. S2. The relative abundance of Bacteria from Phylum(A) and Genus(B) in the Duodenum**

**
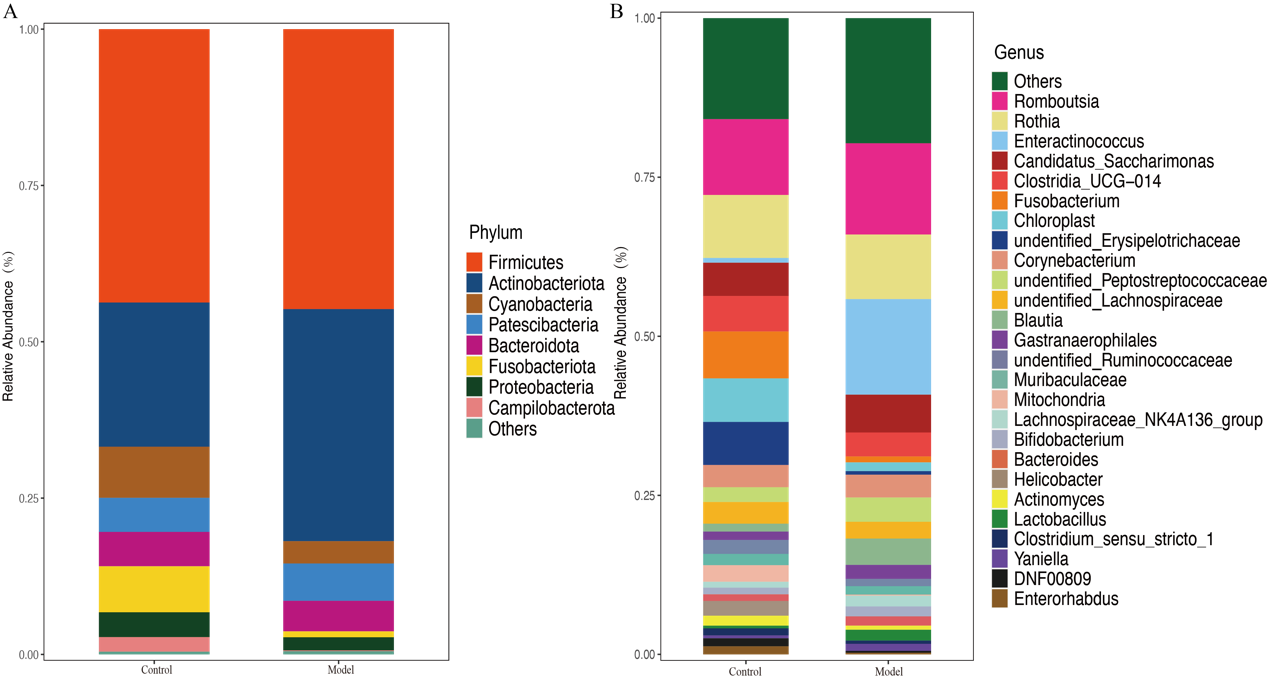
**

**Fig. S3. The relative abundance of Bacteria from Phylum(A) and Genus(B) in the jejunum**

**
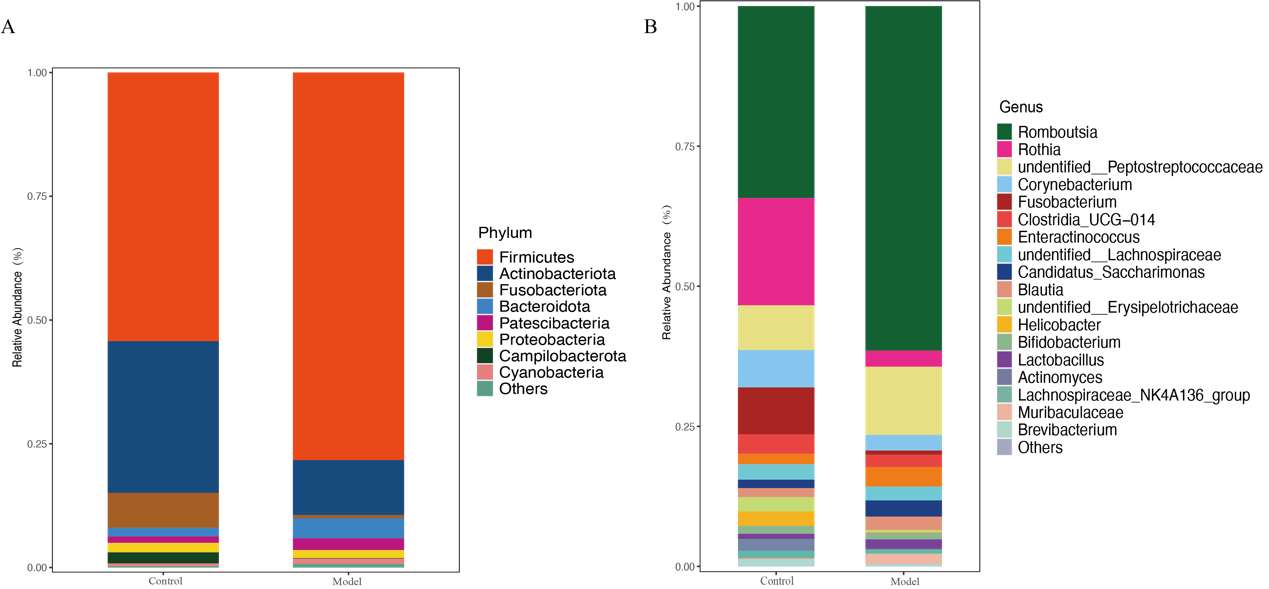
**

**Fig. S4. The relative abundance of Bacteria from Phylum(A) and Genus(B) in the ileum**

**
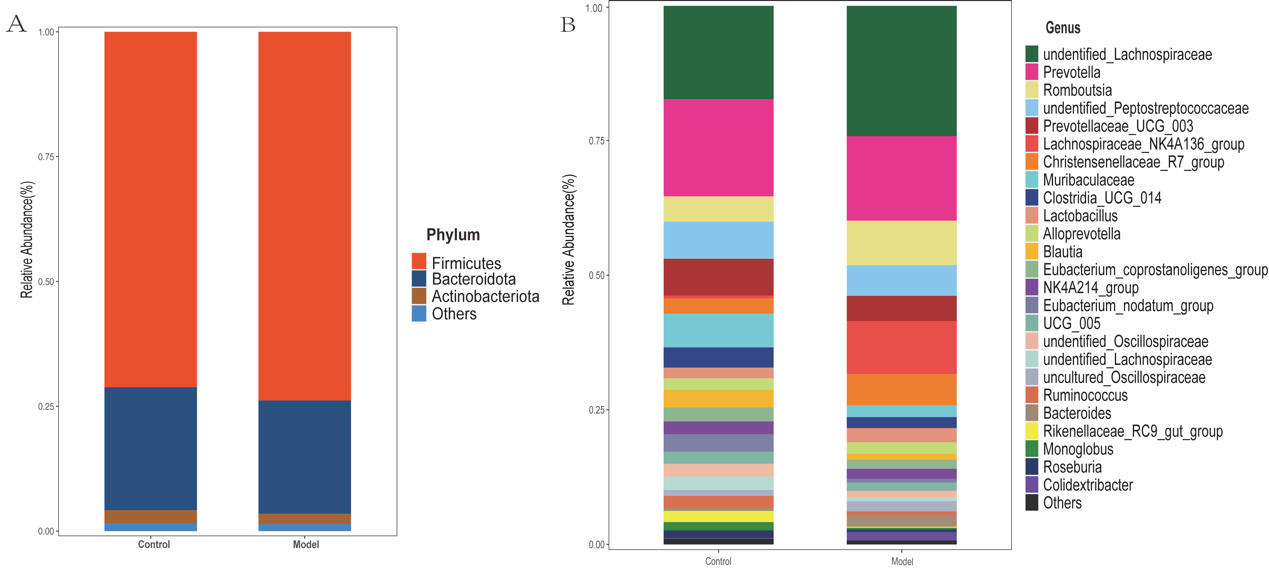
**

**Fig. S5. The relative abundance of Bacteria from Phylum(A) and Genus(B) in the cecum**

**
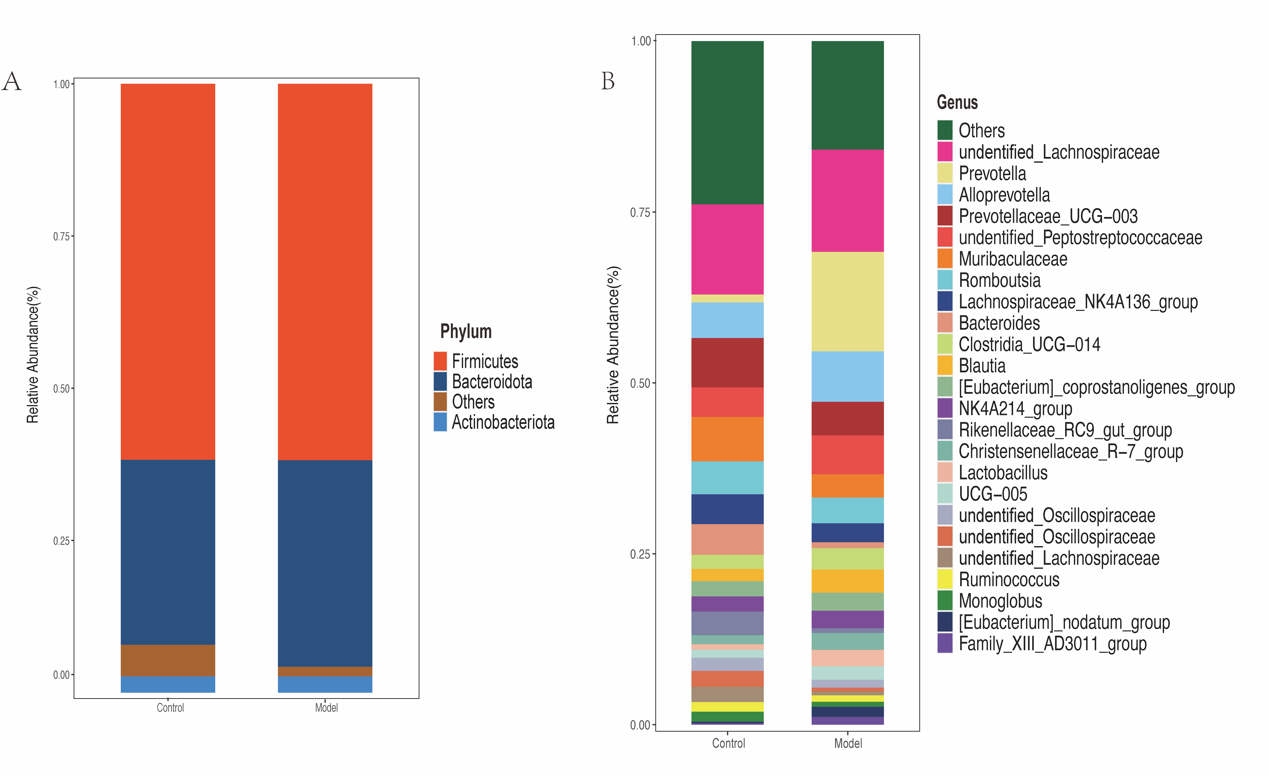
**

**Fig. S6. The relative abundance of Bacteria from Phylum(A) and Genus(B) in the colon**
